# MOTOR PATHWAYS TO DEMENTIA

**DOI:** 10.1093/geroni/igad104.0934

**Published:** 2023-12-21

**Authors:** Joe Verghese

**Affiliations:** Albert Einstein College of Medicine, Bronx, New York, United States

## Abstract

Emerging evidence indicates that non-cognitive manifestations such as gait impairments occur early during dementia. The Motoric Cognitive Risk (MCR) is a pre-dementia syndrome characterized by slow gait and cognitive complaints. MCR is a simple and easily accessible clinical dementia risk assay that does not require cognitive testing. MCR has incremental predictive validity for Alzheimer’s disease and related dementias over its individual cognitive and motoric components. Recent studies from across the world have helped to elucidate the epidemiology, biology, and brain substrates of MCR; paving the way for future interventions to prevent progression to dementia.

